# Myeloid Cells in the Mouse Retina and Uveal Tract Respond Differently to Systemic Inflammatory Stimuli

**DOI:** 10.1167/iovs.62.10.10

**Published:** 2021-08-11

**Authors:** Samantha J. Dando, Renee Kazanis, Paul G. McMenamin

**Affiliations:** 1 Queensland University of Technology, Centre for Immunology and Infection Control, School of Biomedical Sciences, Faculty of Health, Brisbane, Australia; 2 Department of Anatomy and Developmental Biology, Monash University, Clayton, Australia

**Keywords:** retina, uveal tract, microglia, systemic inflammation, antigen presenting cells, myeloid cells, LPS, macrophage

## Abstract

**Purpose:**

In spite of clear differences in tissue function and significance to ocular disease, little is known about how immune responses differ between the retina and uveal tract. To this end we compared the effects of acute systemic inflammation on myeloid cells within the mouse retina, iris-ciliary body, and choroid.

**Methods:**

Systemic inflammation was induced in *Cx3cr1^gfp/gfp^* and *CD11c-eYFP Crb1^wt/wt^*mice by intraperitoneal lipopolysaccharide (LPS). *In vivo* fundus imaging was performed at two, 24, and 48 hours after LPS, and ocular tissue wholemounts were immunostained and studied by confocal microscopy. Flow cytometry was used to investigate the expression of activation markers (MHC class II, CD80, CD86) on myeloid cell populations at 24 hours. For functional studies, retinal microglia were isolated from LPS-exposed mice and cocultured with naïve OT-II CD4+ T-cells and ovalbumin peptide. T-cell proliferation was measured by flow cytometry and cytokine assays.

**Results:**

Systemic LPS altered the density and morphology of retinal microglia; however, retinal microglia did not upregulate antigen presentation markers and failed to stimulate naïve CD4+ T-cell proliferation *in vitro*. In contrast, uveal tract myeloid cells displayed a phenotype consistent with late-activated antigen-presenting cells at 24 hours. Systemic LPS induced remodeling of myeloid populations within the uveal tract, particularly in the choroid, where dendritic cells were partially displaced by macrophages at 24 hours.

**Conclusions:**

The disparate myeloid cell responses in the retina and uveal tract after systemic LPS highlight differential regulation of innate immunity within these tissue environments, observations that underpin and advance our understanding of ocular immune privilege.

Located behind the blood-retina barrier, retinal microglia exist within an “immune-privileged” environment.^1^ Like their brain counterparts, retinal microglia are highly specialized tissue resident macrophages that are derived from yolk sac progenitors.^2^ As the sole resident immune cells of the healthy neural retina, microglia undergo continuous tissue surveillance via their motile processes^3^ and respond to tissue perturbations including inflammation, injury, or infection.^4^ Microglia become “activated” during a range of retinal diseases, including uveitis, age-related macular degeneration, glaucoma, diabetic retinopathy, and retinitis pigmentosa,^5^ and during systemic infection.^6^^,^^7^ During ocular inflammation microglia illustrate their plasticity in function by having both protective^8^ and harmful roles^9^ in different disease settings. In addition to their involvement in disease processes, retinal microglia also contribute to tissue homeostasis and communicate (via neurotrophic factors and receptor-ligand interactions) with photoreceptors, retinal pigment epithelium and Müller cells to maintain retinal health.^10^ In mice, sustained depletion of microglia leads to dystrophic, irregular presynaptic termini in the outer plexiform layer (OPL) and decreased ERG b-wave amplitudes, indicating that retinal microglia play a role in synaptic structure and function.^11^ To further understand the immunological and physiological factors that allow normal retinal function, one needs to appreciate the closely adjacent uveal tract.

The uveal tract, comprising of the iris, ciliary body and choroid, is the melanized and highly vascular supportive layer of the eye. The uveal tract is richly endowed with myeloid cells^12^ similar to the homologous bordering tissues of the brain (meninges and choroid plexus).^13^ Macrophages and dendritic cells (DCs) were first described in the mouse and rat uveal tract in the 1990s^14^^,^^15^; however, these cells received little attention until it was suggested that choroidal macrophages may contribute to the pathogenesis of age-related macular degeneration,^16^^–^^18^ a leading cause of blindness in the developed world. Similar to retinal microglia, and indeed other tissue resident macrophages, choroidal macrophages also play a role in maintaining homeostasis of their tissue environment. Recent work by Yang et al.^19^ demonstrated that depletion of mouse choroidal macrophages using colony stimulating factory 1 receptor (CSF1R) inhibitors (frequently used to deplete microglia^20^^,^^21^) resulted in choroidal vascular atrophy and pathological changes in the retinal pigment epithelium. The role of iris and ciliary body macrophages and DCs in homeostasis and during disease remains largely under investigated to date.

In the brain, the regional tissue microenvironment greatly influences the phenotype and function of immune cells. For example, in response to systemic lipopolysaccharide (LPS) myeloid cells within the meninges function as potent antigen-presenting cells (APCs),^22^ whereas most brain microglia are poor APCs by comparison.^22^^–^^25^ These findings can be attributed to differences in anatomical location relative to the blood-brain barrier, because immune privilege does not extend to the nonneuronal bordering tissues of the brain.^1^ Similarly, it is proposed that the uveal tract that borders the retina does not possess the immunomodulatory properties of the neural retina,^1^^,^^4^ although few studies have directly compared immune responses in the retina, iris-ciliary body, and choroid during ocular inflammation. We currently know that in homeostatic conditions, anatomical location does indeed influence the phenotype and longevity of resident macrophages within the eye. Namely, whereas retinal microglia are long-lived, choroidal macrophages appear to be short-lived monocyte-derived macrophages, and iris-ciliary body macrophages comprise a mixture of long-lived and short-lived cells.^2^ Given these basal differences in resident populations and the discussion presented above relating to meningeal APCs and brain parenchymal microglia, we hypothesized that innate immune responses to pathogenic signals driven by myeloid cells would differ significantly between the neural retina and the uveal tract. Furthermore, we postulated that responses may also differ between the anterior and posterior regions of the uveal tract.

To test these hypotheses, we investigated the effects of systemic inflammation on myeloid populations within the mouse retina and uveal tract. Herein, we report disparate myeloid cell responses in the mouse retina and uveal tract after acute, LPS-induced ocular inflammation. In line with our previous studies of the mouse brain and meninges,^22^^,^^26^ we demonstrate that retinal microglia undergo morphological changes in response to systemic LPS but fail to mature into functional APCs that stimulate naïve CD4 T-cell proliferation. Uveal tract myeloid cells, however, do adopt an activated APC phenotype. We further show basal phenotypic differences in myeloid cell subsets within the healthy iris-ciliary body and choroid but demonstrate that acute macrophage responses to LPS-induced inflammation are similar between these tissue regions. These findings advance our understanding of innate immune responses within the eye and support the proposal that immune privilege is restricted to the neural retina and not its bordering tissues.

## Methods

### Ethics Statement

All manipulations were performed in accordance with the Association for Research in Vision and Ophthalmology Statement for the Use of Animals in Ophthalmic and Vision Research and the National Health and Medical Research Council Australian Code for the Care and Use of Animals for Scientific Purposes. Animal breeding and experimental procedures were approved by the Monash Animal Research Platform Ethics Committee.

### Mice and Systemic Inflammation Model

BALB/c *Cx3cr1^gf^^p/gfp^*, C75Bl/6 *CD11c-eYFP Crb1^wt/wt^*, and OT-II mice were used in this study. In *Cx3cr1^gfp/gfp^* mice, both copies of the fractalkine receptor gene (*Cxc3r1*) gene are replaced with *gfp*, and Cx3cr1-expressing myeloid cells (as well as some subsets of natural killer and T-cells) are GFP+.^27^ In the healthy mouse eye, *Cx3cr1* is robustly expressed by retinal microglia, perivascular macrophages, and uveal tract myeloid cells.^28^ Although *Cx3cr1^gf^^p/gfp^* mice are Cx3cr1 deficient, our laboratory showed that Cx3cr1 deficiency does not affect the morphology, immunophenotype, distribution, and density of GFP+ myeloid cells within the uveal tract and retina,^28^ nor does it affect the response to systemic CpG-oligodeoxynucleotide–mediated inflammation in the retina.^29^ *CD11c-eYFP Crb1^wt/wt^*mice are Cx3cr1 sufficient and contain subpopulations of myeloid cells that express eYFP under the control of the *Itgax* promoter.^26^ We previously identified small numbers of CD11c-eYFP+ cells within the healthy mouse brain and retina and identified these as a subset of microglia.^26^ In this study we used *Cx3cr1^gf^^p/gfp^* and *CD11c-eYFP Crb1^wt/wt^*reporter mouse lines to examine the phenotypic and functional responses of different subsets of ocular myeloid cells to acute systemic inflammation. OT-II mice^30^ on a C57Bl/6 background were used to source naïve CD4+ T-cells for antigen presentation assays.

Mice were bred in specific pathogen-free conditions and were maintained on a 12:12-hour light/dark cycle with access to food and water as desired. Young (8–16 weeks) adult mice (male and female) were used for all experiments.

To induce acute systemic inflammation, *Cx3cr1^gfp/gfp^* and *CD11c-eYFP Crb1^wt/wt^*mice received an intraperitoneal injection of *Escherichia* *coli* OIII:B4 LPS (9 mg/kg, cat. no. L3024; Sigma Aldrich, St. Louis, MO, USA) in a 100 µL volume. Control mice were administered 100 µL of sterile saline solution (vehicle). Previous studies have demonstrated that this dose of LPS induces robust immune activation in the mouse brain and retina.^22^^,^^31^

### *In Vivo* Multimodal Retinal Imaging

To perform fundus examination, mice were anaesthetized by an intraperitoneal injection of ketamine (80 mg/kg) and xylazine (10 mg/kg). The pupils were then dilated with 0.5% tropicamide drops (Mydriacil; Alcon, Geneva, Switzerland), and corneas were kept moist with GenTeal eye gel (Novartis, Basel, Switzerland). Fundus imaging was performed using a Micron III retinal imaging system (Phoenix Research Laboratories, Pleasanton, CA, USA) with StreamPix 5 software. Brightness and contrast adjustments were uniformly applied to images in Adobe Photoshop. Immediately after *in vivo* imaging, mice were euthanized for tissue collection.

### Tissue Collection

Mice were deeply anaesthetized with an overdose of Lethabarb (sodium pentobarbital, 150 mg/kg) and were transcardially perfused through the left ventricle with PBS or PBS followed by 4% (w/v) paraformaldehyde. Eyes were then enucleated and processed either for immunostaining, flow cytometry, or functional studies.

### Wholemount Immunostaining

Eyes from perfusion-fixed mice were fixed for an additional 24 hours in 4% (w/v) paraformaldehyde and then microdissected to obtain wholemounts of retina, iris-ciliary body, and choroid as previously described.^32^ Tissues were incubated in 20 mM EDTA disodium salt (37°C for 30 minutes), washed in 1 × PBS (3 × 5-minute washes) and then blocked in 3.0% (w/v) BSA and 0.3% (v/v) Triton X-100 in 1 × PBS at room temperature for 60 minutes. Wholemounts were then incubated in primary antibodies (either rat anti-I-A/I-E [1:300, cat. no. 556999; BD Bioscience, Franklin Lakes, NJ, USA] or guinea pig anti-Tmem119^33^ [1:1000, cat. no. 400 004; Synaptic Systems, Goettingen, Germany]) overnight at 4°C, washed in 1 × PBS and then incubated in secondary antibody (either goat anti-rat IgG-Cy3 [1:300, cat. no. A10522; Invitrogen, Carlsbad, CA, USA] or goat anti-guinea pig IgG-Alexa Fluor 594 [1:400, cat. no. A11076; Invitrogen) and Hoechst 33342 (1:2000, cat. no. 62249; Invitrogen) for two hours at room temperature. After further washes, tissues were mounted onto microscope slides with ProLong Diamond Antifade Mountant (cat. no. P36961; Invitrogen) and coverslipped.

### Confocal Microscopy and Image Analysis

Wholemount tissues were imaged using a SP5 confocal microscope (Leica Microsystems, Wetzlar, Germany). To calculate the density of retinal Cx3cr1-GFP+ and CD11c-eYFP+ cells within the nerve fiber layer/ganglion cell layer (NFL/GCL), inner plexiform layer (IPL), and OPL, Z stacks through the entire thickness of the retina were captured from three random fields of view per mouse using a x40 (NA 1.25) objective. Maximum intensity projections of the NFL/GCL, IPL and OPL were then generated using Fiji (version 1.53c)^34^ and the number of Cx3cr1-GFP+ cells or CD11c-eYFP+ cells within each of these layers was counted using the “Cell Counter” feature. Cells were defined as being localized to a specific retinal layer based on the layer in which their cell body was located. For each mouse, the total number of GFP+ and YFP+ cells across three fields of view was divided by the total area analyzed to obtain cells/mm^2^. Morphological analysis was performed by Sholl analysis^35^ (Fiji Sholl analysis plugin, version 3.7.0) as described previously^22^^,^^36^ to determine microglia process complexity (calculated by area under the curve analysis of the number of branch intersections at increasing radial distances from the cell body) and ramification index.^37^ For each animal, Sholl analysis was performed on six cells from each layer (NFL/GCL, IPL, and OPL). Cells for Sholl analysis were selected on the basis of the following criteria: (i) not overlapping with other labeled cells (cell body and processes clearly distinguished from neighboring cells) and (ii) all cellular processes located within the field of view. After satisfying these criteria, cells for Sholl analysis were randomly selected from three different fields of view. Mean area under the curve and ramification index values were calculated per mouse.

For iris-ciliary body and choroidal cell density measurements, Z stacks were captured from one field of view per mouse using a x20 (NA 0.7) objective. Cell counts were performed on maximum intensity projection images using the “Cell Counter” feature within Fiji; cell density is expressed as cells/mm^2^.

### Flow Cytometry

Tissues for flow cytometry analysis were obtained from mice that had been transcardially perfused with PBS. Retina, iris-ciliary body and choroid were microdissected from enucleated eyes and pooled from multiple mice for processing. Iris-ciliary body and choroid were cut into small pieces using a blade and incubated in 1.5 mg/mL collagenase IV (cat. no. CLS-4; Worthington Biochemical Corporation, Lakewood, NJ, USA) and 0.4 mg/mL DNase I (cat. no. 10104159001; Roche Diagnostics, Basel, Switzerland) at 37°C for 60 minutes. Retinas did not undergo enzymatic digestion. Tissues were then processed into single-cell suspensions by passing through a 70 µm nylon cell strainer, and cells were quantified using Trypan blue staining. Single-cell suspensions were centrifuged at 500*g* for five minutes, washed twice in ice-cold 1 × PBS and stained with fixable viability dye eFlour 450 (cat. no. 553141; eBiosciences, San Diego, CA, USA) as per the manufacturer's instructions. Cells were then centrifuged (500*g*, five minutes), washed in FACS buffer (0.1% (w/v) BSA, 100 µg/mL DNase I and 3 mM EDTA in PBS, and incubated in Fc Block (cat. no. 553141; BD Bioscience) for 15 minutes at 4°C. Single cell suspensions were stained with fluorophore-conjugated antibodies for 30 minutes at 4°C: rat anti-mouse CD45-BV605 (cat. no. 103140; BioLegend, San Diego, CA, USA), rat anti-mouse I-A/I-E-PerCP-Cy5.5 (cat. no. 562363; BD Bioscience), rat anti-mouse CD86-Alexa Fluor 700 (cat. no. 105024; BioLegend), rat anti-mouse CD11b-APC-Cy7 (cat. no. 557657; BD Bioscience), rat anti-mouse F4/80-PE-Cy7 (cat. no. 123113; Biolegend), Armenian hamster anti-mouse CD11c-BV786 (cat. no. 563735; BD Bioscience) and Armenian hamster anti-mouse CD80-PE-CF594 (cat. no. 562504; BD Bioscience). After staining, cells were washed in FACS buffer and fixed in 2% (w/v) paraformaldehyde for 15 minutes on ice. Samples were run on a Fortessa X-20 flow cytometer (BD Bioscience), and data analysis was performed using FlowJo (version 10.7; Tree Star, Ashland, OR, USA).

### Cell Sorting and Magnetic Isolation

Retinal microglia and spleen CD11c-eYFP+ cells were sorted from *CD11c-eYFP Crb1*^wt/wt^ mice (24 hours after intraperitoneal LPS injection) for antigen presentation assays as previously described.^22^ Microglia were defined as CD45^int^ cells. We further sorted CD45^int^ cells into CD11c-eYFP+ and CD11c-eYFP− microglia subpopulations, which have been phenotypically characterized in our previous study.^26^ Naïve CD4+ T-cells were isolated from OT-II mouse spleens using a CD4 T-cell isolation kit (cat. no. 130-104-454; Miltenyi Biotec, Bergisch Gladbach, Germany) and AutoMACS Pro (Miltenyi Biotec).

### Antigen Presentation Assays

Antigen presentation assays using retinal microglia, CellTrace Violet-labeled naïve OT-II CD4+ T-cells and ovalbumin 323–339 peptide (cat. no. 51023-010; Mimotopes, Melbourne, Australia) were performed as previously described for brain microglia.^22^ T-cell proliferation was assessed by flow cytometry (dilution of CellTrace Violet and upregulation of CD44 expression on T-cells) and multiplex cytokine assays (Bio-Plex Pro Mouse Cytokine 23-Plex Panel, cat. no. M60009RDP; Bio-Rad Life Science, Hercules, CA, USA). We were unable to obtain sufficient numbers of cells from iris-ciliary body and choroid for antigen presentation assays because of the small volume of these tissues in the mouse eye.

### Statistical Analysis

Cell density and microglia morphology data were analyzed using one-way ANOVA with multiple comparisons test or Kruskal Wallis test with multiple comparisons using Graph Pad Prism version 8.2.1 (Graph Pad Software, San Diego, CA, USA). Sample sizes for each experiment are shown in figure legends.

## Results

### Acute Systemic LPS Induces Mild Clinical Changes in the Retina

**Figure 1.**
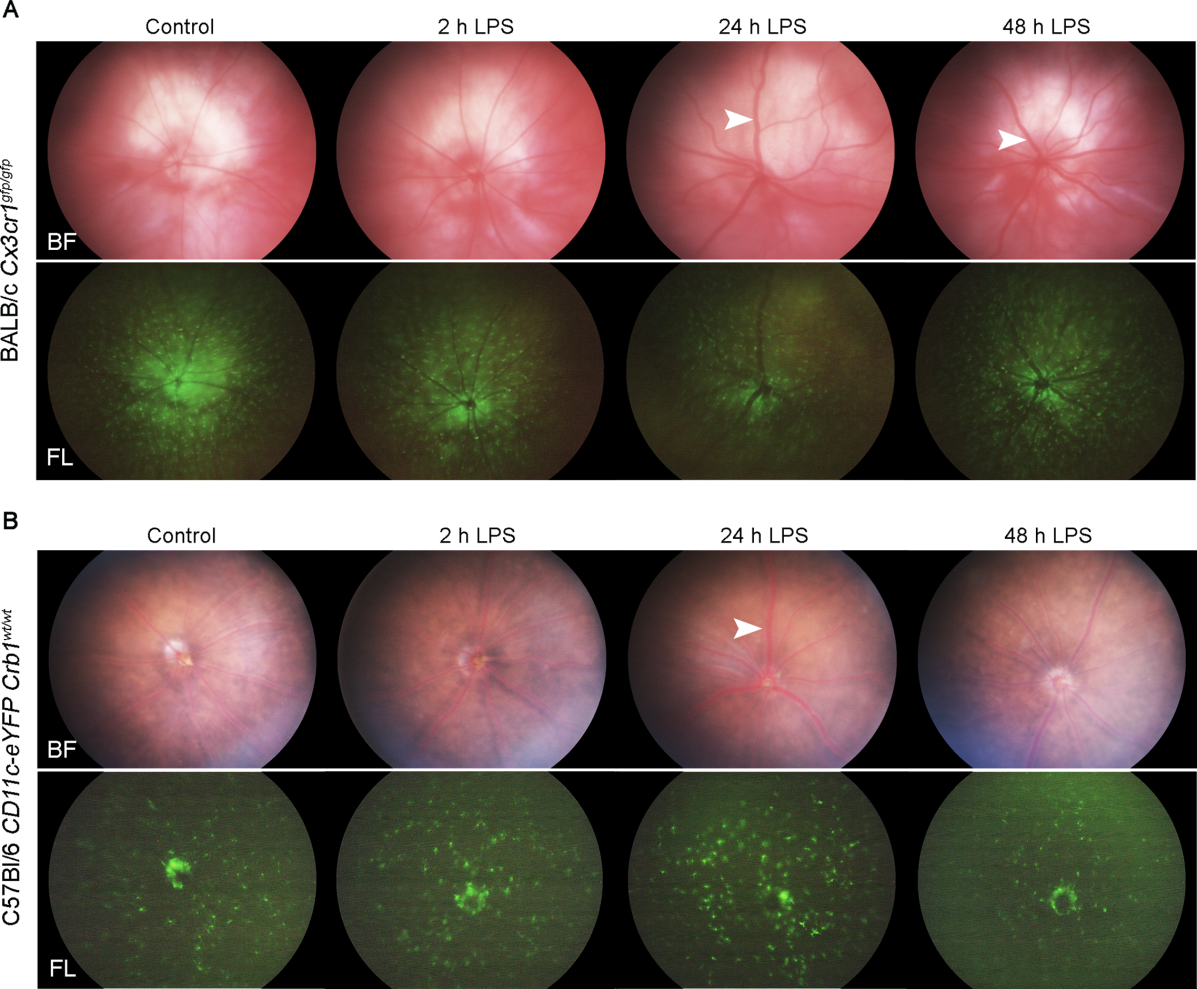
*In vivo* multimodal fundus imaging of reporter mice after acute systemic LPS exposure. Representative fundus images of *Cx3cr1^gf^^p/gfp^* (n = 3-5 mice per time point) and *CD11c-eYFP Crb1^wt/wt^* (n = 3-5 mice per time point) mice. *White arrow heads* show examples of dilated (thickened) vessels; no other significant clinical changes were observed. BF, brightfield; FL, fluorescent.

We initially examined the effects of acute systemic inflammation in the retina using *in vivo* multimodal fundus imaging. Systemic LPS did not induce significant retinal clinical changes in either *Cx3cr1^gf^^p/gfp^* (Fig. 1A) or *CD11c-eYFP Crb1^wt/wt^* (Fig. 1B) mice at two, 24, and 48 hours. We did not observe retinal lesions, atrophy, detachment, or optic disc inflammation at any time point using brightfield imaging. Some thickening of retinal vessels was observed at 24 and 48 hours after LPS exposure; however, vasculitis, perivascular infiltrates, and intravascular infiltrates were absent, suggesting minimal influx of circulating leukocytes at these time points. Although we cannot discriminate resident microglia from infiltrating myeloid cells in these mice solely on the basis of fluorescent reporter expression, the observation that systemic LPS exposure did not result in large accumulations of Cx3cr1-GFP+ cells and CD11c-eYFP+ cells compared to controls also suggested a lack of significant extravasation of circulating myeloid cells at these time points. Taken together, acute systemic LPS exposure in *Cx3cr1^gf^^p/gfp^* and *CD11c-eYFP Crb1^wt/wt^* mice induced only very mild clinical changes in the retina.

### Acute Systemic LPS Exposure Alters Retinal Microglia Density and Morphology

**Figure 2.**
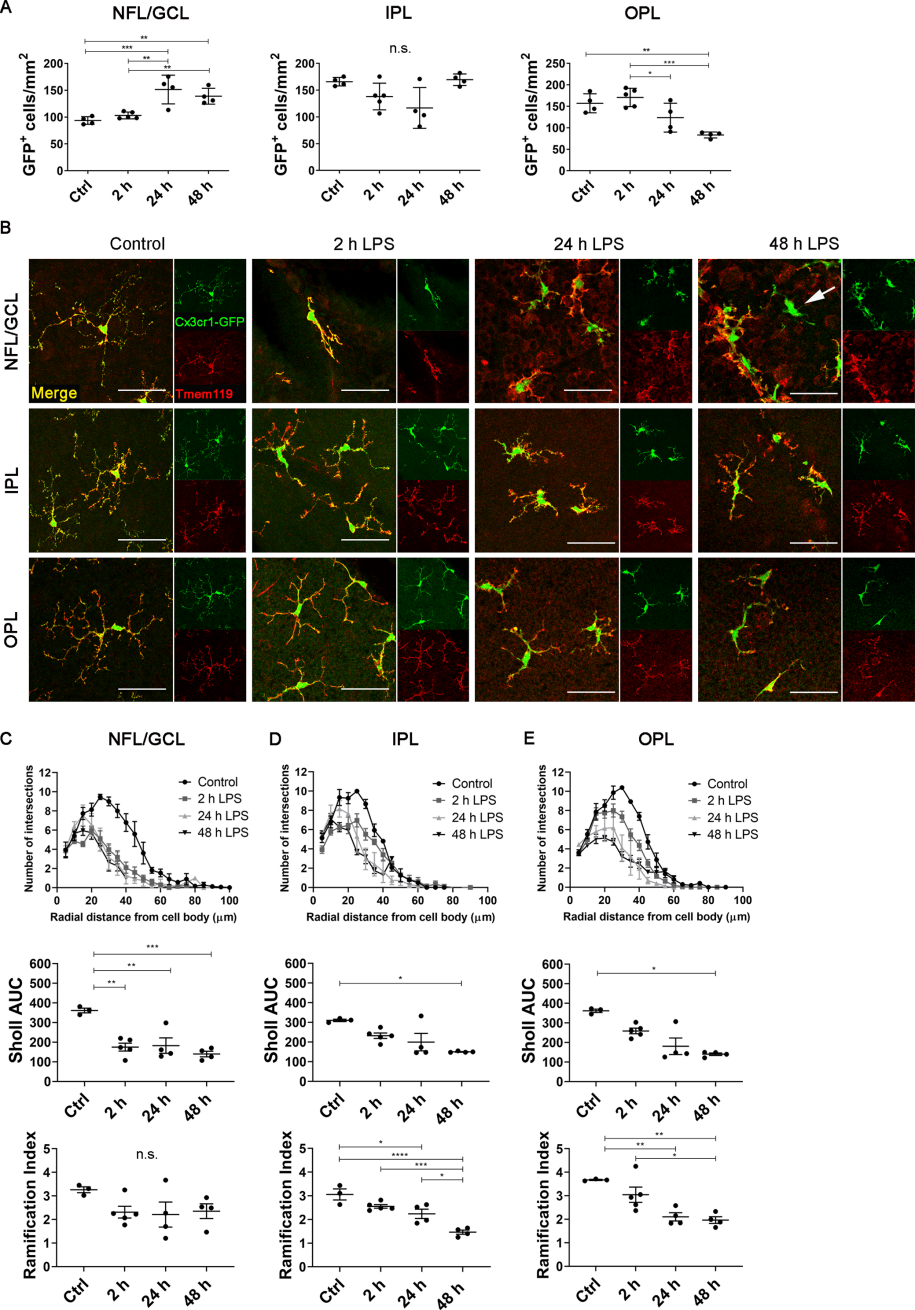
Density and morphological analysis of retinal Cx3cr1-GFP+ microglia. **(A)** Cx3cr1-GFP+ cell density in the NFL/GCL, IPL, and OPL after acute systemic LPS. **(B)** Representative confocal microscopy images of retinal wholemounts stained with Tmem119 from *Cx3cr1^gf^^p/gfp^* mice. Note that all Cx3cr1-GFP+ cells in control, two-hour LPS, and 24-hour LPS mice coexpressed Tmem119, confirming their identity as microglia. Occasional Cx3cr1-GFP+ Tmem119− cells (*arrow*) were observed at 48 hours in the NFL/GCL. *Scale bars:* 50 µm. **(C–E)** Sholl analysis of retinal Cx3cr1-GFP+ microglia demonstrating decreased process complexity (indicated by area under the curve [AUC]) and ramification index after systemic LPS. Data from **(A)** and **(C–E)** were analyzed using either one-way ANOVA or Kruskal Wallis test with multiple comparisons; n = 4-5 mice/group. Individual data points on graphs represent mean data for each mouse, calculated from six cells obtained from three fields of view. n.s., not significant. *P < 0.05; **P < 0.01; ***P < 0.001; ****P < 0.0001.

We used confocal microscopy to determine whether acute systemic LPS affected the density, distribution and morphology of retinal myeloid cells in *Cx3cr1^gf^^p/gfp^* mice. Systemic LPS induced a significant increase in the density of Cx3cr1-GFP+ cells in the NFL/GCL at 24 and 48 hours compared to controls (Fig. 2A). Conversely, the density of Cx3cr1-GFP+ cells was unchanged in the IPL and was significantly reduced in the OPL at 48 hours. All imaged retinal Cx3cr1-GFP+ cells coexpressed the microglia-specific marker Tmem119^33^ in control, two-hour LPS, and 24-hour LPS groups (Fig. 2B). We therefore identified these cells as microglia. Occasional Cx3cr1-GFP+ Tmem119- cells were observed in the NFL/GCL at 48 hours after LPS exposure (Fig. 2B). We assumed that these small numbers of Cx3cr1-GFP+ Tmem119− cells were either infiltrating myeloid cells, perivascular macrophages (if vessel-associated), or microglia that had lost/downregulated Tmem119 expression.

Morphological changes were observed in retinal Cx3cr1-GFP+ microglia as early as two hours after LPS exposure. The morphological changes included classical signs of microglial activation such as thickened cell bodies and processes, decreased process length, and de-ramification (Additional File 1A). Sholl analysis revealed decreased microglial process complexity (as determined by area under the curve analysis) and decreased microglial ramification in each of the retinal layers examined (Figs. 2C–2E), with the earliest significant changes observed in the NFL/GCL (Fig. 2C).

**Figure 3.**
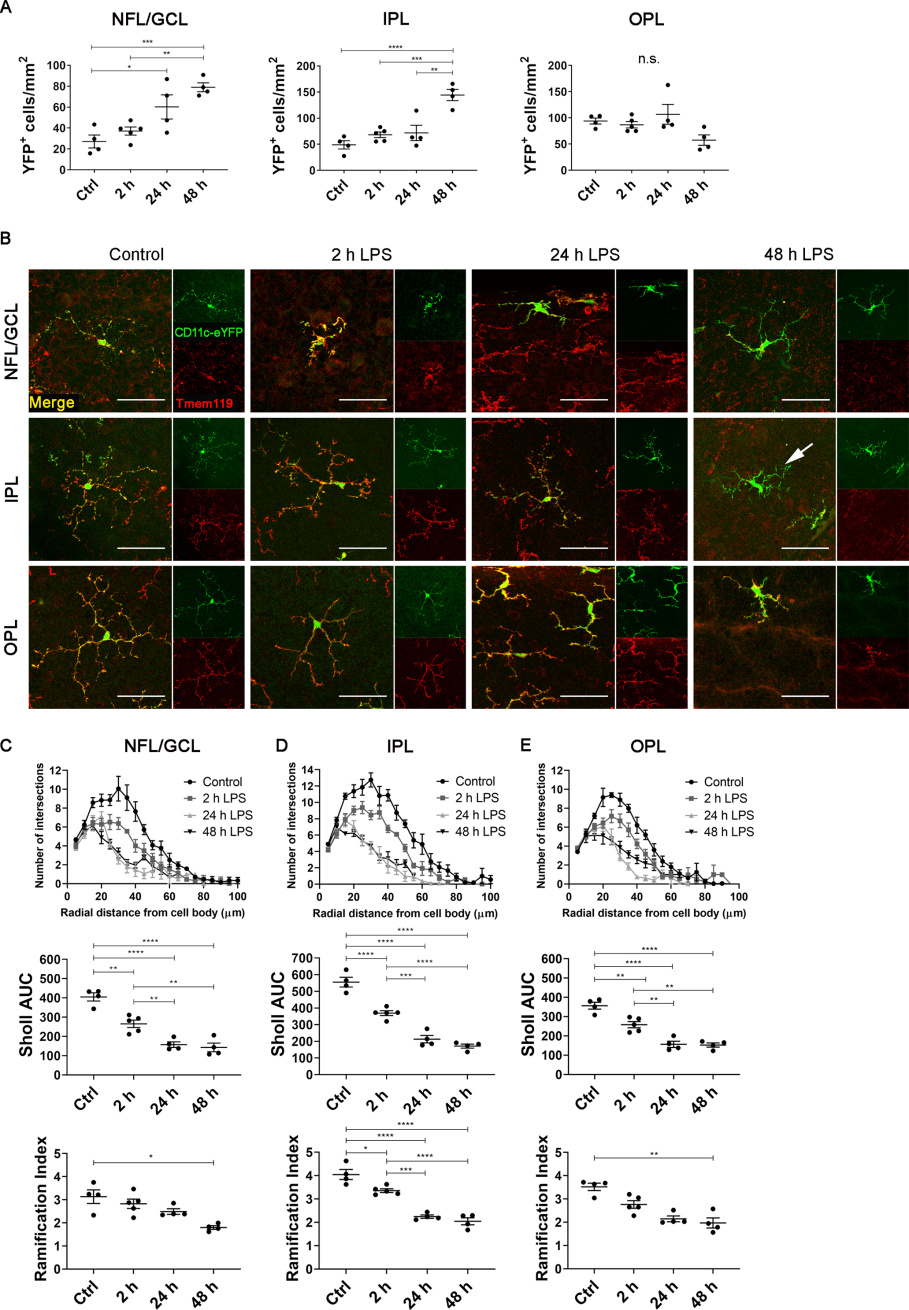
Density and morphological analysis of retinal CD11c-eYFP+ microglia. **(A)** CD11c-eYFP+ microglia density in the NFL/GCL, IPL, and OPL after acute systemic LPS. **(B)** Representative confocal microscopy images of retinal wholemounts stained with Tmem119 from *CD11c-eYFP Crb1^wt^*^/^*^wt^* mice. Note that all CD11c-eYFP+ cells in control, two-hour LPS, and 24-hour LPS mice coexpressed Tmem119, confirming their identity as a subset of microglia. Occasional CD11c-eYFP+ Tmem119- cells (*arrow*) were observed at 48 hours. *Scale bars*: 50 µm. **(C)** Sholl analysis demonstrating decreased CD11c-eYFP+ microglial process complexity (indicated by decreased area under the curve [AUC]) and Schoenen ramification index after acute systemic LPS. Data from **A** and **C–E** were analyzed using one-way ANOVA or Kruskal Wallis test with multiple comparisons; n = 4-5 mice/group. Individual data points on graphs represent mean data for each mouse, calculated from six cells obtained from three fields of view. *P < 0.05; **P < 0.01; ***P < 0.001; ****P < 0.0001.

We next examined the effects of acute systemic LPS on the density, distribution and morphology of retinal CD11c-eYFP+ cells in *CD11c-eYFP Crb1^wt/wt^* mice. Our laboratory previously reported that retinal CD11c-eYFP+ cells are a subset of microglia that are phenotypically indistinguishable from the larger population of YFP− microglia in the healthy retina^26^; however, their response to systemic inflammation has not been investigated. The density of retinal CD11c-eYFP+ cells increased in the NFL/GCL and IPL (but not OPL) after acute systemic LPS (Fig. 3A). Consistent with our observations in *Cx3cr1^gf^^p/gfp^* mice, retinal CD11c-eYFP+ cells from control, two-hour LPS and 24-hour LPS mice co-expressed Tmem119, confirming their identity as bonafide microglia (Fig. 3B). Occasional CD11c-eYFP+ Tmem119− cells were observed in the 48-hour LPS group (Fig. 3B). CD11c-eYFP+ microglia displayed morphological changes associated with microglial activation by two hours after LPS challenge (Additional File 1B). Quantitative Sholl analysis confirmed that CD11c-eYFP+ microglial process complexity and ramification were significantly decreased in LPS-exposed mice (Figs. 3C–3E).

Combined, confocal microscopy analysis demonstrated that acute systemic LPS exposure induced marked changes in the density, distribution, and morphology of retinal microglia in both *Cx3cr1^gf^^p/gfp^* and *CD11c-eYFP Crb1^wt/wt^* mice. These changes were not dependent on genetic background (BALB/c vs. C57Bl/6) or *Cx3cr1* sufficiency.

### Acute Systemic LPS Does Not Mature Retinal Microglia Into Functional Antigen Presenting Cells

**Figure 4.**
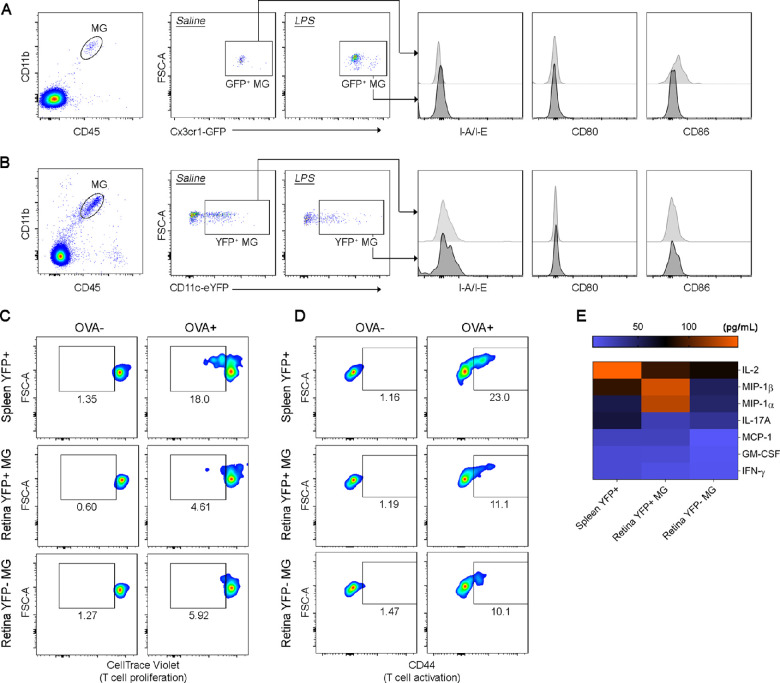
Acute systemic LPS did not mature retinal microglia into functional antigen presenting cells. Comparison of I-A/I-E, CD80, and CD86 expression in retinal microglia (MG) from *Cx3cr1^gf^^p/gfp^* mice **(A)** and *CD11c-eYFP Crb1^wt/wt^* mice **(B)** that received either intraperitoneal saline solution (control) or LPS. Flow cytometry analysis was performed 24 hours after injection. After gating of singlets and viable cells, microglia were gated as CD45^int^ CD11b+. All retinal microglia in *Cx3cr1^gf^^p/gfp^* mice were GFP+ (A); whereas a subpopulation of retinal microglia in *CD11c-eYFP Crb1^wt/wt^* mice were YFP+ **(B)** (as expected). Data representative of results from two independent experiments, retinas from n = 8-17 mice were pooled for each experiment. **(C–E)** Flow cytometric analysis of CellTrace Violet–labeled OT-II CD4+ T-cell proliferation **(C)** and CD44 expression **(D)** after 72 hours coculture with either spleen CD11c-eYFP+ cells, retinal CD11c-eYFP+ MG or retinal CD11c-eYFP− MG (ratio of T-cells to antigen-presenting cells is 33:1) in the presence (OVA+) or absence (OVA−) of ovalbumin 323-339. **(E)** Cytokine and chemokine levels in the supernatants for antigen presentation assays. Numbers in flow cytometry plots indicate the percentage of proliferating CD4+ T-cells (C) or percentage of CD44 expressing CD4+ T-cells (D). Retinal CD11c-eYFP+ and CD11c-eYFP− MG for antigen presentation assays were sorted from pooled cell suspensions from n = 20-24 *CD11c-eYFP Crb1^wt/wt^* mice 24 hours after LPS injection. Naïve CD4+ T-cells were isolated from n = 3 OT-II mice. Data are representative of two independent experiments.

The activated morphology of retinal microglia in LPS-exposed mice led us to hypothesize that acute systemic LPS exposure is sufficient to cause functional alterations in retinal microglia, which may alter their antigen-presenting capability. We therefore examined the expression of MHC class II (I-A/I-E) and the antigen presentation costimulatory markers CD80 and CD86 on retinal microglia 24 hours after systemic LPS injection. Our data revealed that retinal Cx3cr1-GFP+ microglia and CD11c-eYFP+ microglia from control mice did not express I-A/I-E, CD80 or CD86. The expression of these markers was not upregulated on microglia populations 24 hours after systemic LPS injection (Figs. 4A, 4B).

To further explore the possibility that microglia may become capable of antigen presentation in response to systemic LPS exposure, we also performed functional antigen presentation assays using *CD11c-eYFP Crb1^wt/wt^* mice 24 hours after systemic LPS exposure. We reasoned that using *CD11c-eYFP Crb1^wt/wt^* mice for these experiments would have the additional advantage of allowing us to examine whether CD11c-eYFP+ and CD11c-eYFP− microglia subpopulations differ in their response to systemic inflammation, which could have been masked in the phenotypic studies described above. Retinal CD11c-eYFP+ microglia or CD11c-eYFP− microglia were cocultured with naïve OT-II CD4+ T-cells +/− OVA 323-339 for 72 hours. Cocultures containing spleen CD11c-eYFP+ cells instead of microglia served as positive controls, and splenic CD11c-eYFP+ cells induced T-cell proliferation (Fig. 4C) and upregulation of the activation marker CD44 on a subset of T-cells (Fig. 4D). High levels of IL-2 were also detected in culture supernatants containing spleen CD11c-eYFP+ cells (Fig. 4E). In contrast, retinal CD11c-eYFP+ and CD11c-eYFP− microglia failed to stimulate naïve OT-II CD4+ T-cell proliferation (Fig. 4C). Interestingly though, retinal microglia were not inert and did in fact induce CD44 expression in a small population of CD4+ T-cells (Fig. 4D).

Consistent with the lack of T-cell proliferation in assays containing CD11c-eYFP+ and CD11c-eYFP− microglia, lower levels of IL-2 were detected in supernatants from cocultures containing retinal microglia compared to spleen CD11c-eYFP+ cells. However, the chemokines MIP-1α (CCL3) and MIP-1β (CCL4) were detected in higher concentrations in assays containing CD11c-eYFP+ microglia compared to CD11c-eYFP− microglia and spleen CD11c-eYFP+ cells (Fig. 4E). Taken together, these findings showed that (i) acute systemic LPS inflammation was insufficient to mature retinal microglia into effective antigen presenting cells; (ii) although retinal microglia displayed an “activated” morphology, this was not associated with upregulation of antigen-presenting cell activation markers or the ability to stimulate naïve CD4+ T-cell proliferation; and (iii) CD11c-eYFP+ microglia displayed a unique ability to stimulate chemokine production.

### Redistribution of MHC Class II+ Myeloid Cells in the Uveal Tract After Acute Systemic LPS

**Figure 5.**
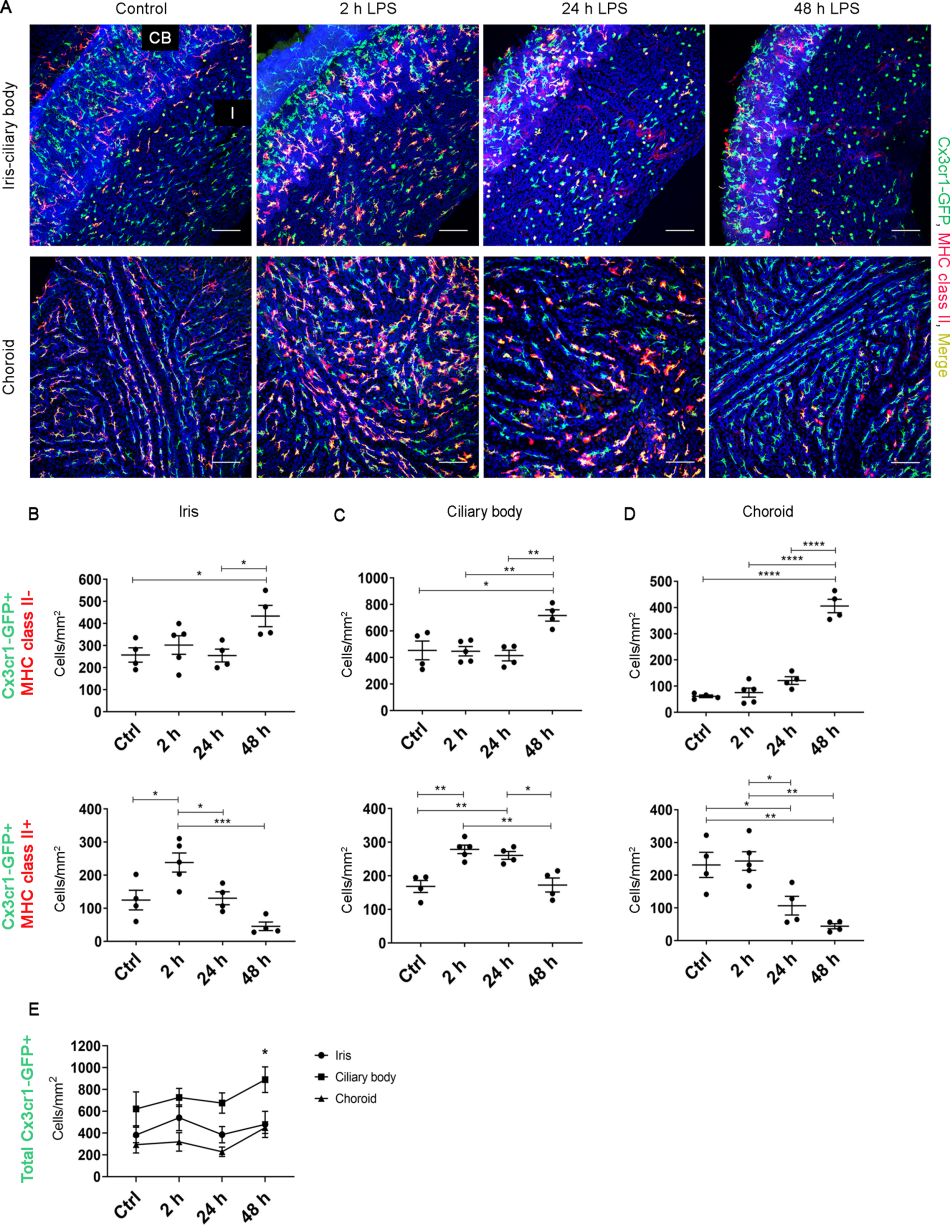
Changes in the density of myeloid cells within the uveal tract after acute systemic LPS. **(A)** Representative confocal microscopy images of iris-ciliary body and choroidal whole mounts from *Cx3cr1^gfp/gfp^* mice that were stained with MHC class II antibodies and Hoechst. I, iris; CB, ciliary body. *Scale bars*: 50 µm. **(B–D)** Changes in the density of Cx3cr1-GFP+ MHC class II + cells and Cx3cr1-GFP+ MHC class II-cells in the iris, ciliary body, and choroid in LPS-exposed mice. **(E)** Density of total Cx3cr1-GFP+ cells in the uveal tract. Asterisk (*) represents a statistically significant increase in total Cx3cr1-GFP+ cells in the ciliary body and choroid (but not iris) at 48 hours compared to controls. Data were analyzed using either one-way ANOVA or Kruskal Wallis test with multiple comparisons; n = 4-5 mice/group. Individual data points on graphs represent mean data for each mouse. *P < 0.05; **P < 0.01; ***P < 0.001; ****P < 0.0001.

Having investigated how systemic inflammation affects myeloid cells within the retina, we next sought to characterize the changes in myeloid cells within the uveal tract. BALB/c *Cx3cr1^gf^^p/gfp^* mice were used to examine the effects of acute systemic LPS on Cx3cr1-GFP+ myeloid cells in the nonpigmented iris, ciliary body and choroid. A large proportion of Cx3cr1-GFP+ myeloid cells in the healthy uveal tract coexpressed MHC class II, and systemic LPS induced a significant shift in the density of both MHC class II+ and MHC class II− myeloid cells (Figs. 5A–5D). In LPS-injected mice, the density of MHC class II+ myeloid cells increased in the iris (Fig. 5B) and ciliary body (Fig. 5C) at two hours but returned to baseline levels by 48 hours. This was associated with a concomitant increase in the density of Cx3cr1-GFP+ MHC class II− cells in the iris and ciliary body at 48 hours.

In the choroid, the density of MHC class II+ myeloid cells was significantly decreased at 24 and 48 hours compared to controls (Fig. 5D). Similar to the iris and ciliary body, this corresponded with a significant increase in the density of MHC class II− myeloid cells in the choroid at 48 hours, by which time Cx3cr1-GFP+ MHC class II− cells were the predominant myeloid cell type within the uveal tract. Despite these changes, the density of total myeloid cells in the iris, ciliary body, and choroid was not different between control and LPS-exposed mice at two hours and 24 hours (Fig. 5E). However, at 48 hours after LPS there was an overall increase in the density of Cx3cr1-GFP+ myeloid cells in the ciliary body and choroid (Fig. 5E). Taken together, these findings demonstrate that systemic LPS induces rapid changes in the proportion of MHC class II+ myeloid cells in the uveal tract.

### Expression of Activation Markers Is Altered on Iris-Ciliary Body and Choroidal Myeloid Cells After Acute Systemic LPS

**Figure 6.**
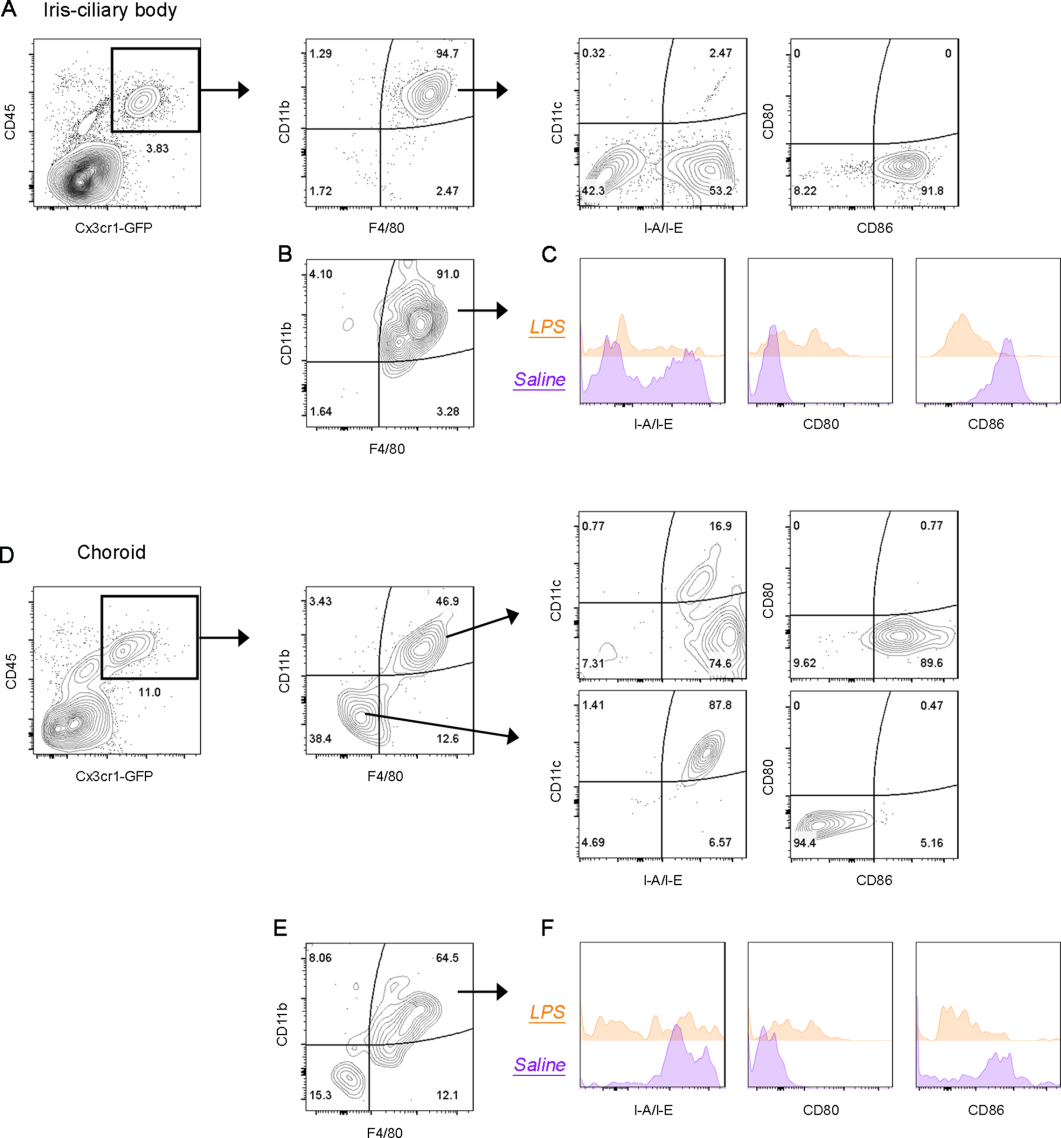
Immunophenotyping of myeloid cells in the uveal tract of healthy and LPS-exposed mice. **(A)** Characterization of Cx3cr1-GFP+ macrophage-like cells in the healthy *Cx3cr1^gfp/gfp^* mouse iris-ciliary body. Twenty-four hours after LPS injection, Cx3cr1-GFP+ cells in the iris-ciliary body maintained a CD11b+ F4/80+ phenotype **(B)**; however, the expression of antigen presentation activation markers (I-A/I-E, CD80, CD86) was altered compared to control (saline) mice **(C)**. **(D)** Two distinct populations (CD11b+ F4/80+ “macrophage-like” and CD11b+F4/80− “cDC-like”) of Cx3cr1-GFP+ cells are present in the choroid of healthy mice. **(E)** Increased percentage of Cx3cr1-GFP+ CD11b+ F4/80+ cells in the choroid 24 hours after LPS exposure. **(F)** Comparison of I-A/I-E, CD80 and CD86 expression by choroidal Cx3cr1-GFP+ CD11b+ F4/80+ cells isolated from control (saline) and 24-hour LPS mice. Data representative of results from two independent experiments; uveal tract tissues from n = 16-25 mice were pooled for each experiment. Data were pre-gated on singlets and live cells.

To further characterize the Cx3cr1-GFP+ myeloid populations within the uveal tract, we performed flow cytometry phenotyping of iris-ciliary body and choroid from control and LPS-exposed mice (24-hour time point). Cx3cr1-GFP+ cells in the iris-ciliary body of control mice displayed a macrophage (CD11b+ F4/80+) phenotype. These macrophages were CD11c− CD80− CD86+ and, consistent with our confocal microscopy data, a subset expressed MHC class II (I-A/I-E+) (Fig. 6A). In LPS-exposed mice, iris-ciliary body Cx3cr1-GFP+ cells also displayed a CD11b+ F4/80+ macrophage phenotype (Fig. 6B); however, I-A/I-E and CD86 expression was decreased, and CD80 expression was increased compared to control mice (Fig. 6C).

Cx3cr1-GFP+ cells in the healthy choroid comprised two distinct populations. Similar to the iris-ciliary body, this included CD11b+ F4/80+ macrophages that were I-A/I-E+ CD86+ CD80−. A subset of these cells also expressed CD11c (Fig. 6D). The second population of choroidal Cx3cr1-GFP+ cells in control mice were CD11b− F4/80− CD11c+ I-A/I-E+ CD80− CD86− (Fig. 6D). Although we did not undertake further immunophenotyping for DCs, this profile is suggestive of classical DCs (cDCs). Twenty-four hours after systemic LPS, there was a shift in the proportion of macrophages and cDC-like cells in the choroid. The percentage of Cx3cr1-GFP+ CD11b+ F4/80+ macrophages increased, whereas the percentage of Cx3cr1-GFP+ CD11b- F4/80- cDC-like cells decreased (Fig. 6E). This is consistent with our confocal microscopy data demonstrating a marked redistribution of myeloid cells within the uveal tract following acute systemic LPS. Examination of antigen presentation markers in LPS-exposed mice revealed that choroidal macrophages decreased expression of I-A/I-E and CD86, and increased CD80 expression at 24 hours (Fig. 6F). We were unable to analyze changes in the expression of these markers in choroidal cDC-like cells as there were too few of these cells at 24 hours for flow cytometry studies.

## Discussion

Immune privileged tissues display attenuated responses to alloantigens because of their highly specialized, tightly regulated immunomodulatory properties that serve to minimize inflammation in tissues with limited capacity for regeneration.^1^^,^^38^^–^^40^ It is well established that the neural retina, similar to the brain parenchyma, is immune privileged.^1^ However, a misconception is that immune privilege applies equally to all tissue compartments within the eye. We posited that immune privilege does not extend to the uveal tract of the eye (homologous to the leptomeninges), which unlike the retina is populated with MHC class II+ cells^14^^,^^15^ and possesses a rich vascular bed within the connective tissue including fenestrated capillaries close to the pigment epithelia in both the ciliary body and choroid.^41^ In support of this hypothesis our present study demonstrated disparate myeloid cell responses in the mouse retina and uveal tract after acute systemic LPS-induced inflammation.

LPS is a TLR4 agonist that is commonly used in experimental models to induce systemic inflammation. When applied systemically it can cause changes to the blood-brain and blood-ocular barriers resulting in neuroinflammation^42^ and endotoxin-induced uveitis,^43^ respectively. Endotoxin-induced uveitis is commonly used as a model of acute anterior ocular inflammation; however, inflammation has also been reported in the posterior compartment of the eye,^44^ although this has been less well studied. Using *in vivo* multimodal fundus imaging, we observed mild thickening/dilation of some retinal vessels at 24 to 48 hours after LPS intraperitoneal administration in both BALB/c *Cx3cr1^gfp/gfp^* and C57Bl/6 *CD11c-eYFP Crb1^wt/wt^* mice. Similar clinical features have been reported after intravitreal administration of LPS in mice,^45^ although this is a far more locally invasive protocol that breaches the blood-ocular barrier. In our study, acute systemic LPS induced rapid morphological activation of resident myeloid cells within the eye but did not induce significant inflammatory infiltrates in the retina and uveal tract. Rosenbaum et al.^46^ reported concordant findings, demonstrating only few infiltrating cells in the eye following intraperitoneal LPS injection despite increased leukocyte rolling and adhesion within the iris vasculature and ocular cytokine production. These authors proposed that the lack of infiltrating cells was likely due to transient LPS-mediated desensitization of circulating leukocytes to chemotactic signals, which could also explain our results. Regardless of the mechanism, acute systemic LPS appears to be a useful model for studying resident immune cell responses in the eye without significant contamination from circulating leukocytes.

Examination of retinal wholemounts two and 24 hours after LPS administration demonstrated that all imaged Cx3cr1-GFP+ and CD11c-eYFP+ cells at these timepoints coexpressed Tmem119. In the brain, Tmem119 expression is restricted to microglia during steady state^33^; however, recent studies have revealed potential caveats associated with this marker and commercially available Tmem119 antibodies, with suggestions that it is downregulated in adult mouse brain microglia after *in vitro* LPS stimulation.^47^ Therefore caution may be appropriate when interpreting Tmem119 staining. Despite this potential limitation, we found that Tmem119 colocalized with all imaged Cx3cr1-GFP+ and CD11c-eYFP+ cells in control mice and at two and 24 hours after LPS, and our flow cytometry data also validated these cells as resident microglia. These findings support our previous study, which identified CD11c-eYFP+ cells in the healthy neural retina as a subset of microglia.^26^

Systemic LPS induced changes in the distribution, density, and morphology of Cx3cr1-GFP+ and CD11c-eYFP+ retinal microglia as early as two hours after LPS injection. These alterations occurred in all three well-described microglia networks throughout the neural retina. The earliest changes occurred in the innermost layers of the retina, where a significant increase in microglia density was observed in the NFL/GCL of both mouse strains at 24 hours. This is likely due to the proximity of microglia within these layers to large post-capillary venules, which are important sites of fluid exchange and leukocyte interactions with the blood-retina barrier during ocular inflammation.^48^ The increase in NFL/GCL microglia density may be a result of local microglia proliferation or migration of microglia from other retinal layers. Further imaging studies may be required to resolve the spatiotemporal dynamics of these cells.

Changes in cellular morphology have long been considered hallmarks of microglial “activation”; however, whether morphological changes correlate with alterations in microglial function remains unclear. Microglia from the healthy neural retina are poor antigen-presenting cells,^49^ and this is a fundamental tenet of retinal immune privilege. However, in some disease states, such as experimental autoimmune uveitis, microglia upregulate MHC class II expression and genes associated with antigen processing and presentation.^50^ In the current study, both CD11c-eYFP+ and CD11c-eYFP− microglia isolated from 24-hour LPS-injected mice failed to upregulate antigen presentation (I-A/I-E) and costimulatory (CD80 and CD86) markers and were unable to stimulate naïve CD4+ T-cell proliferation. Despite this lack of T-cell proliferation in APC assays, these retinal CD11c-eYFP+ and CD11c-eYFP− microglia from LPS-exposed mice did induce upregulation of CD44 (an activation marker) on a small percentage of T-cells, indicating that microglia could potentially interact with naïve CD4 T-cells. Carson et al.^23^ suggested that adult mouse brain microglia are capable of stimulating naïve T-cell differentiation into Th effector cells without stimulating T-cell proliferation, which could explain our findings in retinal microglia. However, analysis of supernatant cytokines did not suggest that Th polarization occurred in our study. A more detailed flow cytometry analysis of T-cell phenotypes is required to confirm this. Although our results indicate that retinal microglia are not entirely quiescent in APC assays and display morphological signs of activation, their lack of expression of antigen presentation markers and the absence of T-cell proliferation strongly indicate that retinal microglia even after systemic LPS exposure still display weak antigen-presenting capacity. This is in keeping with current concepts of the immune privileged nature of the neural retina and suggests that although retinal microglia are highly responsive to systemic inflammation, they have a very high threshold for maturing into functional immunogenic APCs. Our findings thus continue to support previous work suggesting that ocular immune privilege is maintained during high-grade LPS inflammation^51^ and that retinal microglia may have an immunoregulatory role.^49^

Close parallels with our previous study of cortical microglia^22^ were found when we compared retinal CD11c-eYFP+ and CD11c-eYFP− microglia in APC assays. Similar to cortical CD11c-eYFP+ microglia, we found that retinal CD11c-eYFP+ microglia (but not the larger CD11c-eYFP− retinal microglia population or splenic APCs) displayed a unique ability to produce high levels of MIP-1α (CCL3) and MIP-1β (CCL4). These potent chemoattractants produced by macrophages in response to LPS exposure contribute to monocyte recruitment and retinal degeneration and uveitis in rodent models.^52^^,^^53^ Enhanced chemokine production by CD11c-eYFP+ microglia suggests that these cells are a functionally distinct subpopulation of retinal microglia.

In contrast to the retina, myeloid cells within the uveal tract exhibited phenotypic characteristics of conventional APCs. Immunostaining of wholemount tissues showed significant changes in the density of MHC class II+ and MHC class II− myeloid cells in the iris, ciliary body, and choroid. In line with published studies of the rat uveal tract,^54^^,^^55^ the density of MHC class II+ cells rapidly increased in the iris and ciliary body after systemic LPS injection. As the density of total Cx3cr1-GFP+ cells remained unchanged at two and 24 hours after LPS, it is unlikely that infiltrating myeloid cells or proliferating resident cells significantly contributed to the total pool of Cx3cr1-GFP+ myeloid cells at these time points. Rather, it is likely that the shift in the proportion of MHC class II–expressing cells was due to upregulation of MHC class II by resident myeloid cells. By 48 hours, Cx3cr1-GFP+ MHC class II− cells were the predominant myeloid cell type in each region of the uveal tract. At this timepoint, there was also an increase in total Cx3cr1-GFP+ cells in the ciliary body and choroid, which suggests that infiltrating or proliferating cells may have contributed to the population of Cx3cr1-GFP+ MHC class II− myeloid cells. Fate mapping studies are required to further investigate this, because a limitation of using Cx3cr1-GFP reporter mice is that it is not possible to distinguish circulating GFP+ cells from resident cells.

Using flow cytometry, we further investigated the phenotype of Cx3cr1-GFP+ cells and demonstrated basal differences in Cx3cr1-GFP+ myeloid cell populations within the uveal tract. The healthy iris-ciliary body contained macrophages (F4/80+ CD11b+), whereas the choroid was populated with both macrophages and cDC-like (F4/80− CD11b− CD11c+ MHC class II+) Cx3cr1-GFP+ cells. These data support previous studies that identified macrophages and putative DCs in steady-state rodent choroidal wholemounts.^14^^,^^15^^,^^56^ We anticipated there may be different responses to systemic LPS in the anterior and posterior uveal tract because of differences in homeostatic myeloid cell populations in these regions, as well as heterogeneous leukocyte migration dynamics in the vascular beds of the iris and choroid.^57^ However, macrophage-like cells in both the iris-ciliary body and choroid displayed a similar phenotype that was consistent with late-activated APCs at 24 hours. A subset of iris-ciliary body and choroidal macrophage-like cells upregulated the activation marker CD80 24 hours after LPS, indicating they were mature APCs. Conversely, the expression of CD86 and MHC class II was dramatically decreased at this time point. Decreased MHC class II expression has been reported in late-activated APCs after LPS stimulation,^58^ which is consistent with our findings. Furthermore, professional APCs dynamically regulate CD86 via ubiquitination depending on their need to promote or restrict T-cell activation.^59^ Thus it is possible that reduced MHC class II and CD86 expression by uveal tract APCs is an immunoregulatory mechanism to control T-cell activation, although this requires further investigation.

Combined, our data suggest that similar to the bordering tissues of the brain,^22^ the mouse uveal tract contains APCs and may be an immunological interface between the immune-privileged compartments of the eye and the periphery. A limitation of our study was that we were unable to isolate sufficient numbers of cells from the mouse iris-ciliary body and choroid to perform functional antigen presentation assays; however, a previous study demonstrated that myeloid cells in the rat iris are capable of stimulating T-cell proliferation.^60^ A question for future studies will be whether the observed differences between retinal microglia and uveal tract myeloid cells are primarily due to their microenvironment (immune privileged vs. non-immune privileged ocular environment), ontogeny (yolk sac vs. a combination of yolk sac/bone marrow derived), or other programmed factors. Interestingly, transplantation of non-central nervous system macrophages into the Csf1r−/− mouse brain demonstrated that brain microglial identity is shaped by both the central nervous system environment and cell origin^61^; adapting this approach to study how the tissue environment influences ocular macrophages would shed further light on our findings.

## Conclusions

In summary, retinal microglia undergo marked morphological changes in response to acute systemic LPS but do mature into functional antigen-presenting cells that stimulate naïve CD4+ T-cell proliferation. Thus, even during a systemic inflammatory challenge, tight immunoregulation of microglial responses and immune privilege is maintained within the retina to some extent. In contrast, myeloid cells within the uveal tract respond to systemic LPS in a manner that is consistent with conventional APCs from peripheral tissues. Our study highlights disparate myeloid cell responses in the retina and uveal tract after systemic inflammation and suggests that immune privilege is restricted to the neural retina and not its bordering tissues. These findings have implications for our understanding of immune-mediated diseases of the eye.

## Supplementary Material

Supplement 1
