# Supplementary material for: Myeloid Cells in the Mouse Retina and Uveal Tract Respond Differently to Systemic Inflammatory Stimuli
Source: Invest Ophthalmol Vis Sci. 2021 Aug 11;62(10):10. doi: 10.1167/iovs.62.10.10 (PMC8363776; doi:10.1167/iovs.62.10.10)
Supplement: Supplement 1 [file iovs-62-10-10_s001.pdf]

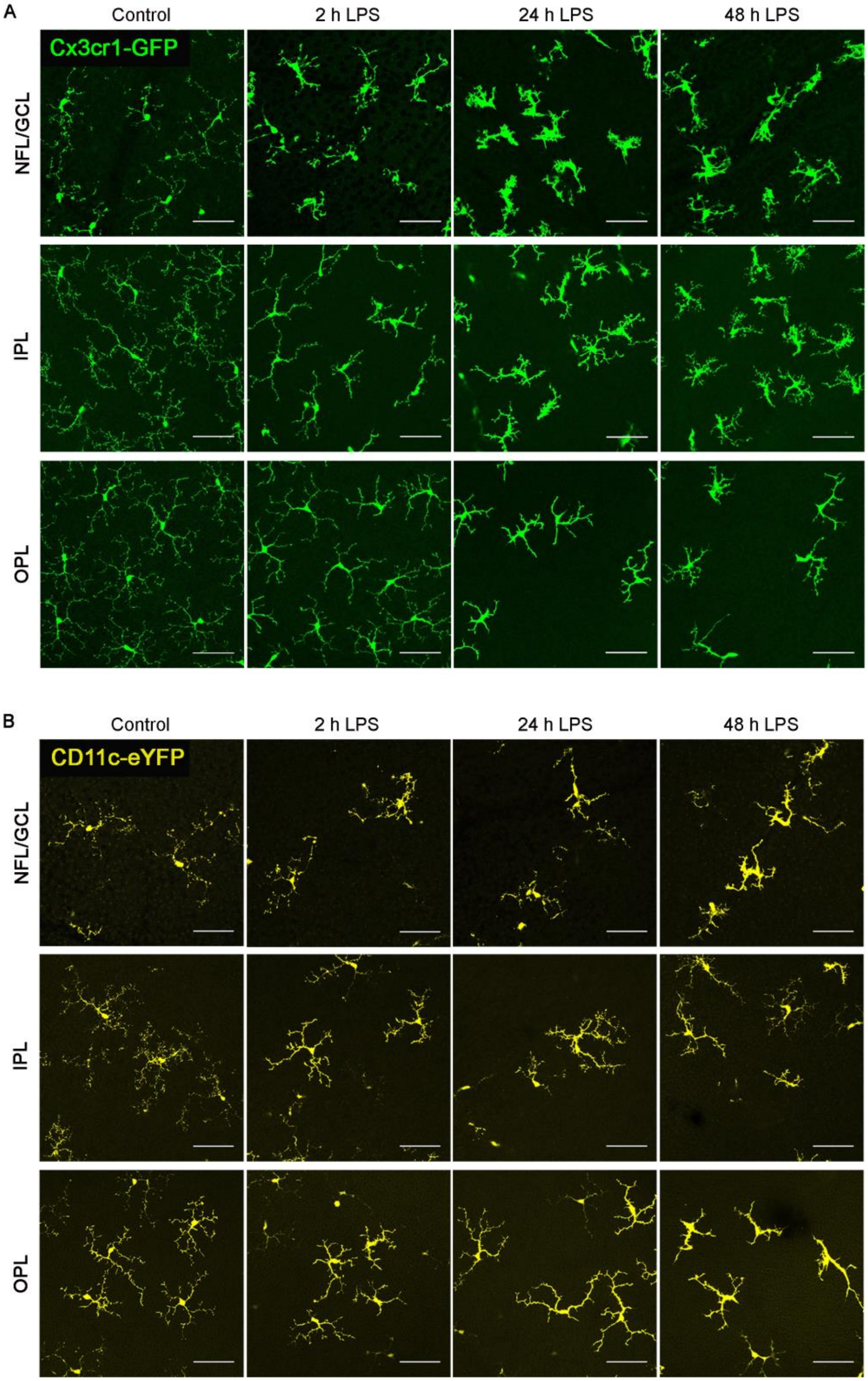

Additional file 1

Additional File 1: Representative confocal microscopy images demonstrating morphological changes (process and cell body thickening, decreased process length and de-ramification) of Cx3cr1-GFP+ microglia **(A)** and CD11c-eYFP+ microglia **(B)** following acute systemic LPS.
